# Delayed Recognition of Type 1 Sigmoid-Colon Atresia: The Perforated Web Variety

**Published:** 2010-08-14

**Authors:** Ghulam Mustafa, Bilal Mirza, Zahid Bashir, Afzal Sheikh

**Affiliations:** Department of Pediatric Surgery, The Children's Hospital and the Institute of Child Health Lahore, Pakistan

**Keywords:** Colonic atresia, Colonic stenosis, Perforated colonic web, Intestinal obstruction

## Abstract

Colonic atresias are the rare malformations of the colon and constitute about 1.7 to 15% of all gastrointestinal (GI) atresias. A 6-month old infant presented with recurrent episodes of sub-acute intestinal obstruction since birth. During the index admission, patient had clinical signs of complete intestinal obstruction. The patient was operated and type I sigmoid-colon atresia found which on further exploration tuned out to be of perforated mucosal web variety. The resection of the involved part of colon and a primary end to oblique colo-colic anastomosis was performed.

## INTRODUCTION

There are three anatomical types of colonic atresia, the less frequent being type I. This is characterized by a mucosal diaphragm, completely occupying the lumen, without sero-muscular interruption [1]. At times this web has a small opening because of which complete obstruction does not occur. This results in delayed recognition of this anomaly with life threatening consequences. Presented here is one such infant in whom this anomaly was not picked up promptly.

## CASE REPORT

A 6-month-old male infant presented to emergency room with abdominal distension, bilious vomiting and constipation for two days. There was a history of repeated episodes of such events since birth. On clinical examination the patient was febrile, with temperature of 100F, pulse 120/min and respiratory rate 28/min. Abdomen was distended with visible bowel loops. Bowel sounds were absent. Digital rectal examination revealed empty rectum. Abdominal radiograph showed air fluid levels (Fig. 1). Nasogastric tube was passed and intravenous fluids and antibiotics started and surgery planned.

**Figure:**
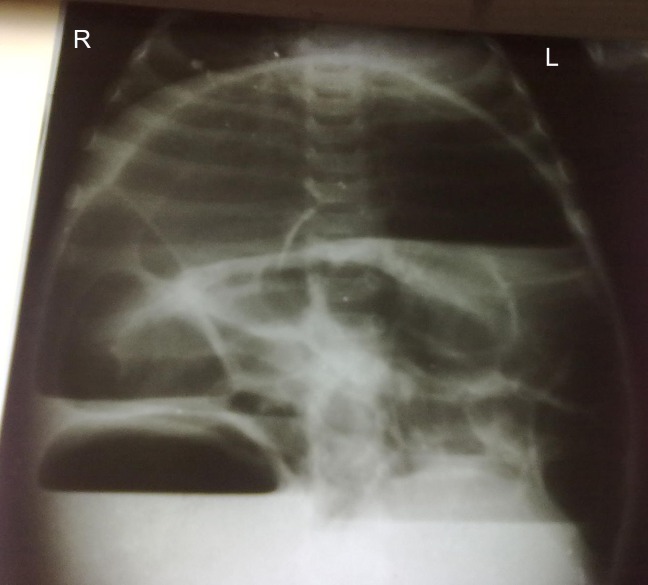
Figure 1: Xray abdomen erect posture showing air fluid levels indicating intestinal obstruction.

At laparotomy dilated sigmoid colon found. On tracing it further distally a narrowed segment reached (Fig. 2). On longitudinal enterotomy over the narrowed area, a web found which had an opening in its center. The margins of the colon over the web and few centimeters of colon around the stenosed area were inflammed and tear easily. This portion of colon was resected and an end to oblique primary colo-colic anastomosis performed. 

**Figure:**
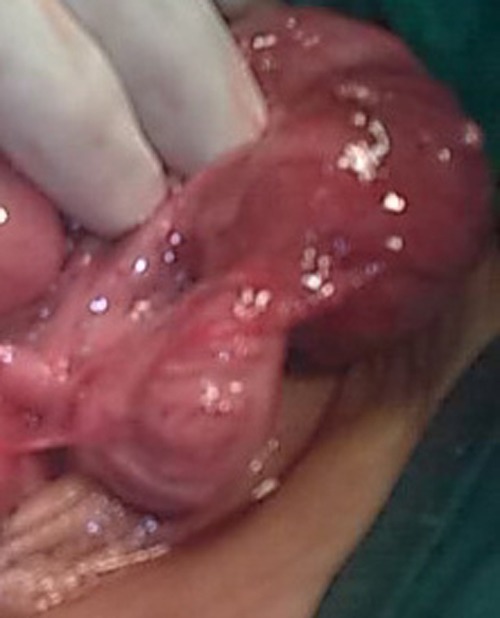
Figure 2: Operative picture showing a distended colon proximal to the web.

The post operative recovery was uneventful. Naso-gastric tube was removed on 4th post operative day and patient started orally. On 7th post-operative day patient was discharged. The histopathology of the specimen revealed inflammation and presence of ganglion cells. Patient remained well at 6 months follow up.

## DISCUSSION

Gut atresias are commonly found in small intestine and colon is a rare site for this anomaly. The reported incidence of colonic atresia is 1 in 20,000 live births. In a series of 277 patients over a period of 25 years, Vecchia et al, reported colonic atresia in 8% cases only [1 , 2 , 3].

The most commonly accepted theory of development of intestinal atresia is based on vascular accidents occurring during the course of fetal development and this mechanism is supposed to be the most probable etiology of the colonic atresia and stenosis [1].

There are three types of colonic atresia. Type I atresia is characterized by a diaphragm inside the lumen and the serosal surfaces of intestine, proximal and distal to atresia, are uninterrupted. Type III colonic atresia is the most frequently reported type [1 , 4]. In our case the there was a web (type I) which was perforated at the centre.

The preoperative diagnosis of colonic atresia and stenosis can be made accurately without the help of various sophisticated diagnostic modalities. Abdominal radiograph may show non-specific intestinal obstruction and in about 10% of cases pneumoperitoneum may occur. Any newborn with a suspicion of intestinal atresia on clinical grounds and plain radiograph findings should have a contrast enema to delineate the unused colon and ascertain any colonic atresia or stenosis. In case of colonic atresia the contrast will typically fill the lumen of distal unused colon, terminating at a point adjacent to the segment that is most distended with luminal air - the cutoff point [1 , 3 , 5 , 6 , 7]. The age of our patient was 6-month thus possibility of colonic atresia was ruled out though stenosis was not suspected. In this case mechanical obstruction of the small intestine was the pre-operative diagnosis.

Various types of surgical procedures are adopted for this anomaly depending upon anatomical location, type of atresia and condition of the baby, though primary repair without any covering stoma is recommended in all types. Due to its association with Hirschsprung’s disease a biopsy of the distal colon and rectum has been advised in all cases. The experience of local coloplasty technique in such cases is very limited and thus not recommended yet [1 , 4 , 5].

A review of literature revealed only one case of congenital colonic stenosis that presented in the infantile age (4-month) [6]. In our case, the patient presented in acute obstruction at 6 months of age though he had recurrent episodes of intestinal obstructions since birth that was not investigated properly. A timely recognition of symptoms would have reduced prolonged morbidity.

## Footnotes

**Source of Support:** Nil

**Conflict of Interest:** None declared
